# NMR-Based Analysis of Plasma Lipoprotein Subclass and Lipid Composition Demonstrate the Different Dietary Effects in ApoE-Deficient Mice

**DOI:** 10.3390/molecules29050988

**Published:** 2024-02-24

**Authors:** Cheng-Hung Yang, Yu-Hsuan Ho, Hsiang-Yu Tang, Chi-Jen Lo

**Affiliations:** 1 Department of Biomedical Sciences, College of Medicine, Chang Gung University, Taoyuan City 33302, Taiwan; chyang@gap.cgu.edu.tw (C.-H.Y.); hokylie4@gmail.com (Y.-H.H.); tangshyu@mail.cgu.edu.tw (H.-Y.T.); 2 Metabolomics Core Laboratory, Healthy Aging Research Center, Chang Gung University, Taoyuan City 33302, Taiwan; 3 Clinical Metabolomics Core Laboratory, Chang Gung Memorial Hospital, Taoyuan City 33302, Taiwan

**Keywords:** NMR, lipoprotein subclasses, ApoE-deficient mice

## Abstract

Plasma lipid levels are commonly measured using traditional methods such as triglycerides (TG), high-density lipoprotein (HDL), low-density lipoprotein (LDL), and cholesterol (CH). However, the use of newer technologies, such as nuclear magnetic resonance (NMR) with post-analysis platforms, has made it easier to assess lipoprotein profiles in research. In this study involving ApoE-deficient mice that were fed high-fat diets, significant changes were observed in TG, CH, free cholesterol (FC), and phospholipid (PL) levels within the LDL fraction. The varied proportions of TG in wild-type mice and CH, FC, and PL in ApoE^-/-^ mice were strikingly different in very low-density lipoproteins (VLDL), LDL, intermediate-density lipoprotein (IDL), and HDL. This comprehensive analysis expands our understanding of lipoprotein subfractions and the impacts of the APOE protein and high-fat diet in mouse models. The new testing method allows for a complete assessment of plasma lipids and their correlation with genetic background and diet in mice.

## 1. Introduction

As cardiovascular diseases become the leading cause of worldwide mortality, the different strains of mice as animal models have been used to study the process of atherosclerosis, one of the primary risk factors of coronary artery disease. Among various mouse models [1,2,3], apolipoprotein-E-deficient (ApoE^-/-^) mice exhibit reduced clearance of lipoprotein metabolism and therefore accumulate cholesterol (CH) levels in the blood circulation. ApoE-knockout mice show a significant increase in total plasma cholesterol, compared to wild-type mice [4]. Furthermore, feeding ApoE^-/-^ mice with a high-fat diet additionally increases plasma CH levels and accelerates atherosclerosis development [5]. Owing to the similarity of lesion distribution to humans [6], ApoE^-/-^ mice are commonly used for relevant atherosclerotic studies, such as on complications of smoking [1].

Atherosclerosis is the pathological basis of cardiovascular disease (CVD), and low-density lipoprotein (LDL) plays a crucial role in the initiation and progression of atherosclerotic cardiovascular disease (ASCVD) [7]. Abundant evidence has demonstrated the causal role of LDL in the development and progression of ASCVD [8,9]. As LDL cholesterol (LDL-C) is the traditional biochemical marker of LDL, the measurement plays a pivotal position in all international guidelines [9,10]. Nevertheless, many patients who have achieved recommended LDL-C levels are still at risk of ASCVD. Numerous prospective studies have reported that LDL particle (LDL-P) measurement is a more precise indicator than LDL-C in the prediction of CVD risk. LDL-Ps vary in size, density, and chemical composition. Clinical studies have substantiated that the atherogenic mechanisms of LDL-Ps are determined not only by the number and size of LDL particles, but also by the modification of LDL. Of note, small, dense LDL particles (sdLDL) have a greater atherogenic propensity, compared to large and intermediate LDL subfractions.

Despite the unequivocally causal role of LDL-C in the development of atherosclerosis, a significant proportion of patients without elevated LDL-C develop ASCVD at long-term follow-up into old age [9]. This indicates that some patients may display detrimental LDL-C. Plasma lipids usually are characterized by conventional measurements of triglycerides (TG), HDL-cholesterol, and LDL-C [11]. It cannot discriminate against the detrimental LDL. Lipoprotein subfractions provide more information than conventional lipid measures that may help improve risk stratification and understand the pathophysiology of cardiovascular disease [12]. Although the understanding of the pathophysiological pathways of the disease highlights the major role of lipoprotein particles, the complexity of lipoprotein particles and the multiple approaches and methodologies report critical issues when used for quantification [13]. However, the gold standard for lipoprotein profile analysis, which ultracentrifugation has been regarded as, is time consuming and cannot deal with large numbers of samples [14]. Thus, clinical practice has primarily focused on overall changes for certain parameters, such as TG and CH, instead of lipoprotein subfraction, due to utility limitations [15].

Recent technology has led to the application of advanced lipoprotein analyses, including the subfraction of lipoprotein profiles, which have been applied to several diseases in research. These methods have gained acceptance in the field of CVD risk assessment and have proven their clinical relevance [13]. The NMR assessment identified several lipoprotein derangements in LDL-Ps among the T1DM (Type 1 diabetes mellitus) population that were overlooked in the conventional analysis [16], and the NMR determined that small HDL subtractions were inversely associated with incident CVD and improved risk stratification for T2D (Type 2 diabetes) adverse outcomes [17]. Although further studies are needed to elucidate their roles in different lipoprotein subfractions in the development of CVD, the information of the lipoprotein profile provides understanding of the efficiency of lipid metabolism in human bodies. Therefore, acquiring these data can be applied to clinical diagnosis, therapy, and medication. Furthermore, it is promising for the development of precision medicine.

As a result, we want to develop a fast, precise, and stable method with low-volume samples via NMR and acquire lipoprotein profiles from its application software and in vitro diagnostic for research in the lipoprotein subclass analysis platform, which provides quantification data of different lipoprotein subfractions and its components in animal studies. In the plasma, chylomicrons and very low-density lipoproteins (VLDL) utilize APOE as their main ligand for clearance from the circulation [18]. This protein has been identified as a promising target for the creation of atherosclerosis models early on [19,20]. For altered content or functionality of APOE in mice, it showed predicted effects on APOB lipoprotein levels and metabolism [21]. In ApoE^-/-^ mice, atherosclerosis development is initiated spontaneously, even when animals are kept on a regular rodent diet, but it can be accelerated by applying a fat-enriched diet [20]. In order to verify the feasibility of the cutting-edge method in lipoprotein analysis, we applied this developed method to investigate the effects on ApoE^-/-^ mice with a high-fat diet in the experiment.

## 2. Results

### 2.1. Global Analysis Demonstrated That High-Fat Diet Affected Different Lipoprotein Subfraction Changes in ApoE^-/-^ and Wild-Type Mice

**Figure 1:**
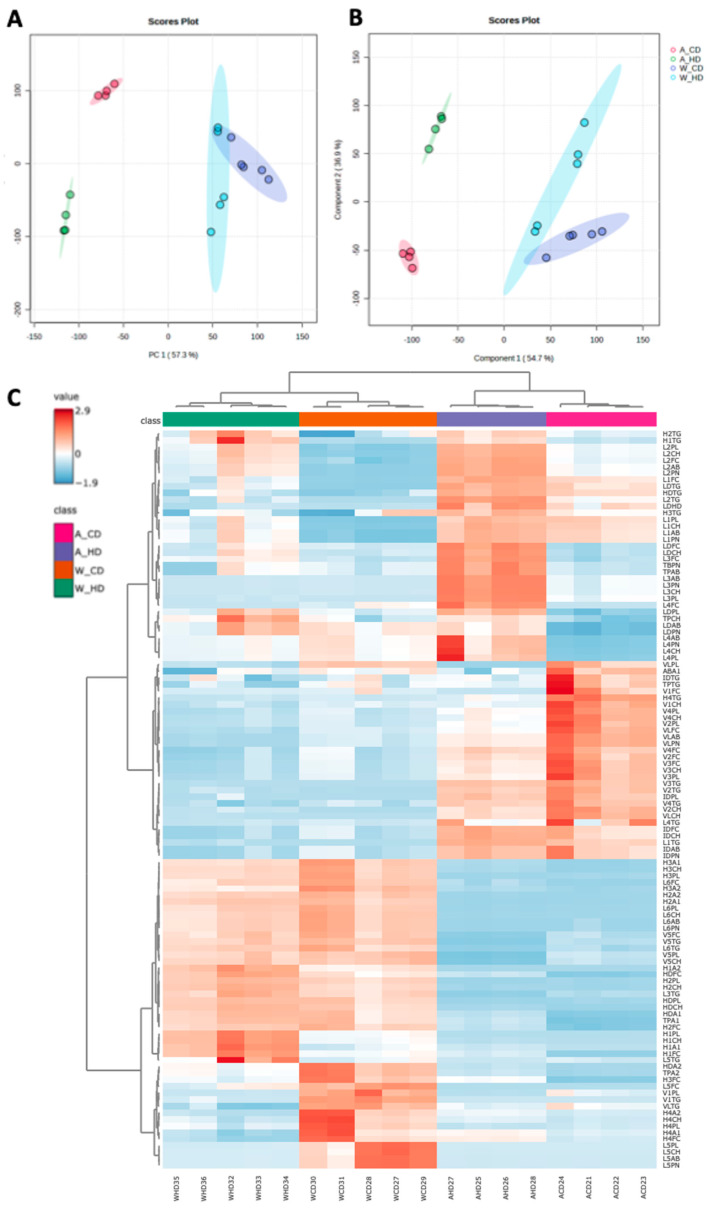
The lipoproteins profiles in ApoE^-/-^ and wild-type mice fed with a high-fat diet. (**A**) The score plots of principal component analysis (PCA), (**B**) partial least squares discriminant analysis (PLSDA), and (**C**) heatmap demonstrate the differences between 112 parameters in four groups of mice, including ApoE^-/-^ mice with a chow diet (A_CD, pink), ApoE^-/-^ mice with a high-fat diet (A_HD, purple), wild-type mice with a chow diet (W_CD, orange), and wild-type mice with a high-fat diet (W_HD, green).

The wild-type mice and ApoE^-/-^ mice were bred for the first eight weeks. From the ninth week, mice were divided into four groups, including ApoE^-/-^ mice with the chow diet (A_CD), ApoE^-/-^ mice with the high-fat diet (A_HD), wild-type mice with the chow diet (W_CD), and wild-type mice with the high-fat diet (W_HD). To clarify the effect of the high-fat diet, we observed the change in body weight in wild-type and ApoE^-/-^ mice between different ages (Supplementary Figure S1). Finally, mice were sacrificed at the end of the twenty weeks, and their plasma samples were analyzed through the NMR spectrometer. In the NMR in vitro diagnostic for research in the lipoprotein subclass analysis platform, we obtained 112 parameters from the lipoprotein profiles (Supplementary Table S1), including the amounts of Apo-A and Apo-B in HDL and LDL, respectively, components in different lipoprotein subfractions, and particle numbers of lipoproteins, except for HDL. In Figure 1A, the principal component analysis (PCA) showed that two types of mice had different distributions. However, they showed a considerable separation in principal component 2 (PC2) in mice fed with different diets. In Figure 1B, the partial least squares discriminant analysis (PLSDA) showed a similar distribution with PCA. In Figure 1C, the heatmap depicts the levels of the lipoprotein class and subclass in four groups of mice, which clearly showed clustering of the same type of mice. Two wild-type mice groups showed differences in lipoprotein profiles between the chow and high-fat diets. The group of wild-type mice with high-fat diets resulted in several lipoprotein subclass changes compared to chow diets, but the changes in lipoprotein profiles were not exactly the same as the ApoE^-/-^ mice. The following charts present the proportion of TG, free cholesterol (FC), CH, and phospholipid (PL) in different lipoprotein classes and subclasses, which clearly pointed out the overall detail changes among four groups of mice.

### 2.2. The Abundance and Distribution of Lipids in the Lipoprotein Class Were Distinct in High-Fat-Treated ApoE^-/-^ and Wild-Type Mice

**Figure 2:**
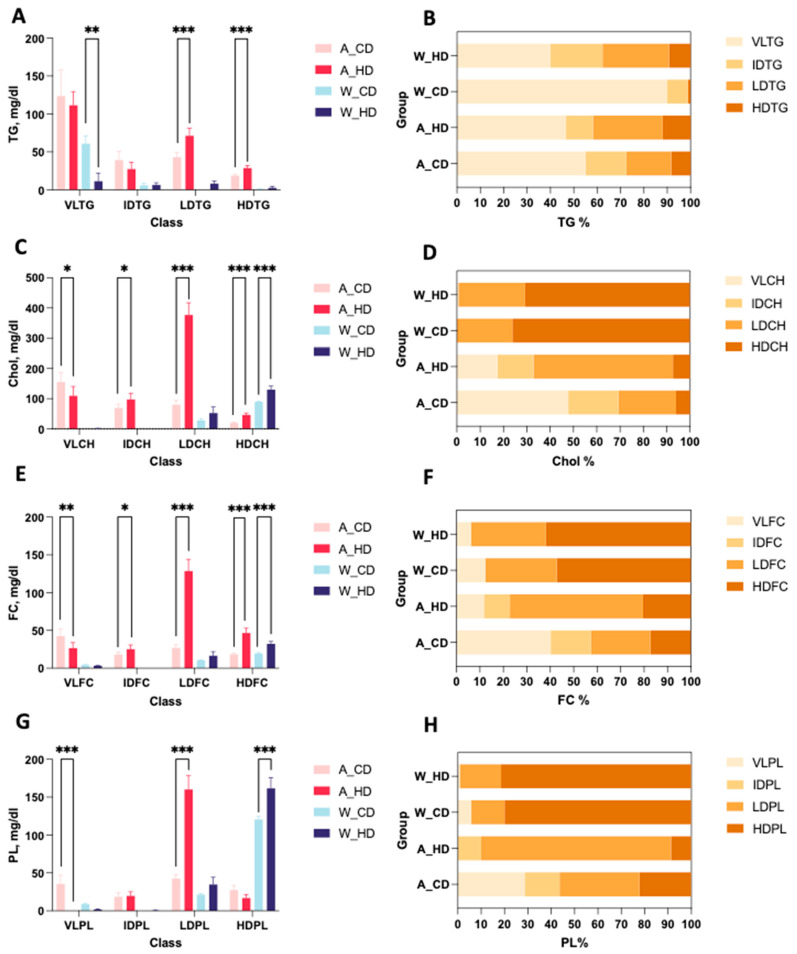
Concentration and proportions of TG, CH, FC, and PL levels in lipoproteins of ApoE^-/-^ mice and wild-type mice with a chow diet and a high-fat diet. There were four groups of mice: ApoE^-/-^ mice with a chow diet (A_CD, pink), ApoE^-/-^ mice with a high-fat diet (A_HD, red), wild-type mice with a chow diet (W_CD, light blue), and wild-type mice with a high-fat diet (W_HD, dark blue). (**A**) The plasma levels of triglyceride (TG) in VLDL, IDL, LDL, and HDL are represented as VLTG, IDTG, LDTG, and HDTG. (**C**) Cholesterol (Chol) in VLDL, IDL, LDL, and HDL is represented as VLCH, IDCH, LDCH, and HDCH. (**E**) Free cholesterol (FC) in VLDL, IDL, LDL, and HDL is represented as VLFC, IDFC, LDFC, and HDFC. (**G**) Phospholipid (PL) in VLDL, IDL, LDL, and HDL is represented as VLPL, IDPL, LDPL, and HDPL. The proportions of TG (**B**), Chol (**D**), FC (**F**), and PL (**H**) in lipoproteins are shown. The asterisks indicate the levels of significance in the same types of mice with different diets. One asterisk (*) indicates the *p*-value < 0.05; two (**) asterisks indicate the *p*-value < 0.01; and three asterisks (***) indicate the *p*-value < 0.001.

In general, two types of mice showed distinct proportions from the components in lipoprotein classes. The bar charts display the TG, CH, FC, and PL levels in the plasma lipoprotein of four groups of mice. Wild-type mice had higher HDL significance than the rest of the lipoprotein classes in bar charts of CH (Figure 2C), FC (Figure 2E), and PL (Figure 2G). On the other hand, ApoE^-/-^ mice had higher LDL significance than the rest of the lipoprotein classes in bar charts of TG (Figure 2A), CH (Figure 2C), FC (Figure 2E), and PL (Figure 2G). Furthermore, the stacked bar charts showed the percentages of TG, CH, FC, and PL in lipoprotein classes, respectively, which can directly present the differences of proportions in four groups of mice. Wild-type mice had similar trends for the distribution of components, except for TG, in lipoprotein classes. On the other hand, two groups of ApoE^-/-^ mice showed different proportions; the group with the high-fat diet had a more varied distribution than the chow diet, which took up over half of the CH, FC, and PL in LDL, especially accounting for 80% of PL.

### 2.3. The Abundance and Distribution of Lipids in the VLDL, LDL, and HDL Subclasses Were Distinct in High-Fat-Treated ApoE^-/-^ and Wild-Type Mice

**Figure 3:**
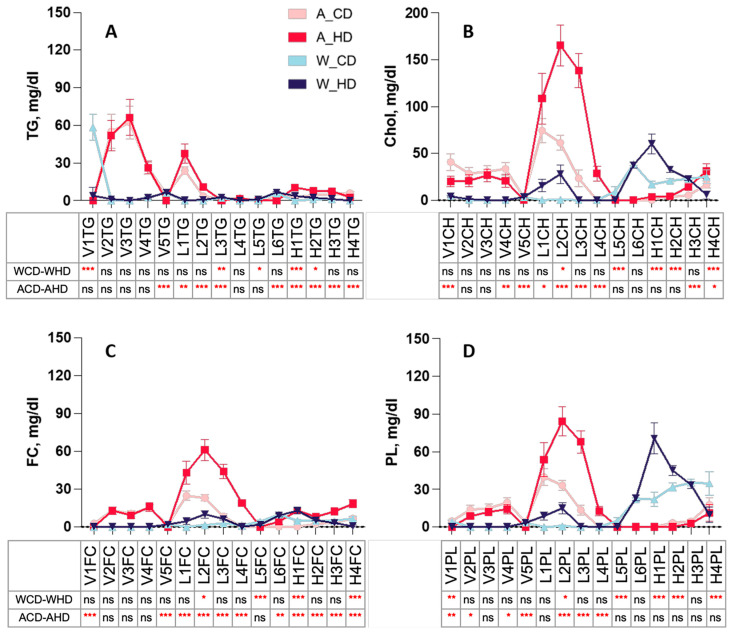
Concentration of TG, CH, FC, and PL levels in plasma VLDL, LDL, and HDL subclasses of ApoE^-/-^ mice and wild-type mice with a chow diet and a high-fat diet. Four groups of mice, ApoE^-/-^ mice with a chow diet (A_CD, pink), ApoE^-/-^ mice with a high-fat diet (A_HD, red), wild-type mice with a chow diet (W_CD, light blue), and wild-type mice with a high-fat diet (W_HD, dark blue), displayed the concentrations in different lipoprotein subclasses, including VLDL, IDL, LDL, and HDL, with the following tables showing the significance between the same type of mice with different diets. (**A**) Triglyceride (TG) in the VLDL subclass (VLTG1-VLTG5), LDL subclass (L1TG-L6TG), and HDL subclass (H1TG-H4TG). (**B**) Cholesterol (Chol) in the VLDL subclass (V1CH-V5CH), LDL subclass (L1CH-L6CH), and HDL subclass (HDL-1-H4CH). (**C**) Free cholesterol (FC) in the VLDL subclass (V1FC-V5FC), LDL subclass (L1FC-L6FC), and HDL subclass (H1FC-H4FC). (**D**) Phospholipid (PL) in the VLDL subclass (V1PL-V5PL), LDL subclass (L1PL-L6PL), and HDL subclass (H1PL-H4PL). The asterisks indicate the levels of significance in the same types of mice with different diets in the following table. One asterisk (*) indicates the *p*-value < 0.05; two (**) asterisks indicate the *p*-value < 0.01; three asterisks (***) indicate the *p*-value < 0.001; ns indicate not significant.

The linear charts provide detailed information on different distributions from the components in each lipoprotein subclass, which displayed TG, CH, FC, and PL levels in the plasma lipoprotein subclass of four groups of mice. Wild-type mice had similar significance in VLDL, LDL, and HDL subclasses, except for H2CH and V1PL of CH (Figure 3B), FC (Figure 3C), and PL (Figure 3D) in the lipoprotein subclass linear chart. Furthermore, HDL displayed distinguished traits with high significance, compared to other lipoproteins, in wild-type mice fed with a high-fat diet.

**Figure 4:**
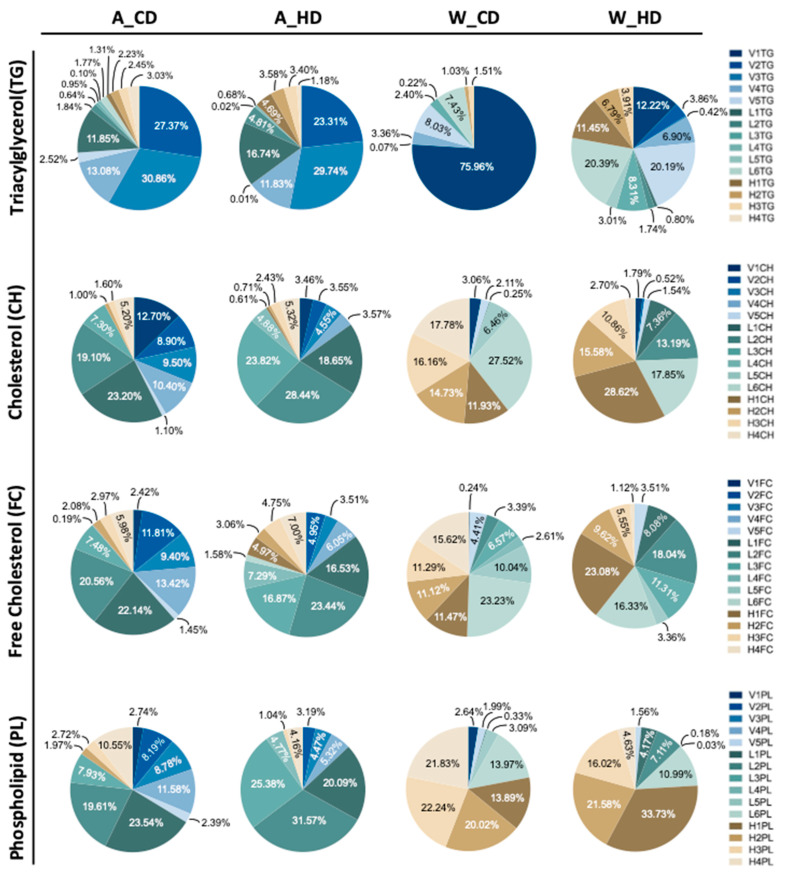
The proportions of TG, CH, FC, and PL levels in the plasma VLDL, LDL, and HDL subclasses of ApoE^-/-^ mice and wild-type mice with chow diets and high-fat diets. Four groups of mice, ApoE^-/-^ mice with a chow diet (A_CD), ApoE^-/-^ mice with a high-fat diet (A_HD), wild-type mice with a chow diet (W_CD), and wild-type mice with a high-fat diet (W_HD), displayed the concentration in different lipoprotein subclasses.

Instead, ApoE^-/-^ mice had similar significances in the VLDL, LDL, and HDL subclasses, except for V1FC and L4FC of triglyceride (Figure 3A) and FC (Figure 3C) in the lipoprotein subclass linear chart. Additionally, among the lipoproteins, most LDL and HDL had high significance. The pie charts show the percentages of TG, CH, FC, and PL in lipoprotein classes, respectively, presenting the differences in each component in four groups of mice (Figure 4). In the group with the chow diet, the mice had the highest CH and FC levels in HDL4 among the HDL subclass. On the contrary, the group with the high-fat diet had the amounts of subfractions decreased by the order of the HDL subclass; HDL1 took up 20~30% of the highest levels, including H1CH, H1FC, and H1PL, which was reflected in the concentration of the linear charts. However, the wild-type mice had large amounts of triglycerides in VLDL in the group with the chow diet, which had 75%, and the group with the high-fat diet had roughly 60% FC in LDL.

On the other hand, ApoE^-/-^ mice had large amounts of components, except for TGs, in LDL, where the groups with a chow diet and a high-fat diet took up half of the CH, FC, and PL, respectively, especially the group with the high-fat diet, which had 75% CH, 65% FC, and 80% PL. However, those mice had large amounts of TG in VLDL, where the group with chow diets had 73% and high-fat diets had 64%.

Furthermore, the difference between the two groups was the proportion of components, except for triglycerides, in LDL. The group with the chow diet had amounts of LDL subfractions decreased by the order of the subclass; LDL1 and LDL4 had the highest and lowest levels, respectively. However, the group with the high-fat diet distinctly showed a broad distribution in LDL, especially LDL2, which took up 20~30% of the highest levels among the LDL subclass, including L2CH, L2FC, and L2PL, which reflected the largest concentration of the lipoprotein class in linear charts.

### 2.4. The Abundance and Distribution of Apolipoprotein in LDL and HDL Subclasses in High-Fat-Treated ApoE^-/-^ and Wild-Type Mice

**Figure 5:**
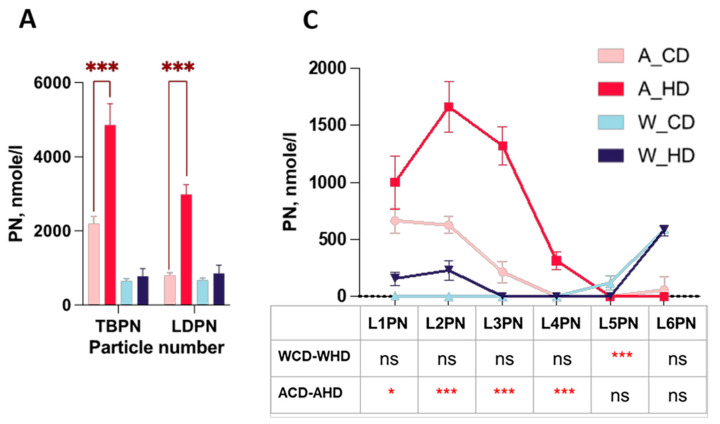

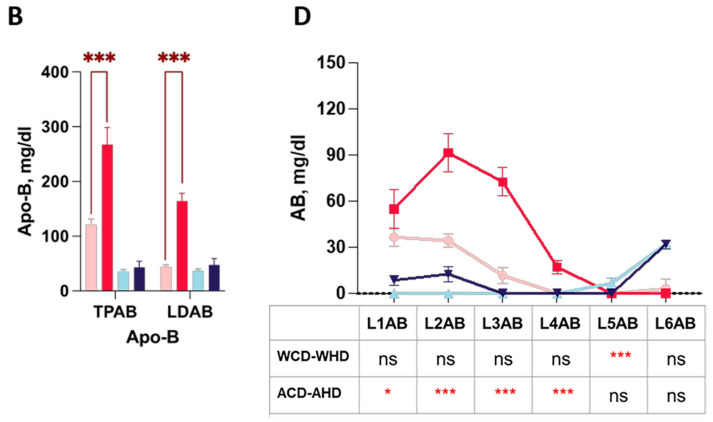
The distributions of the particle number and Apo-B in the LDL subclass in high-fat-treated ApoE-/- and wild-type mice. Four groups of mice, ApoE^-/-^ mice with the chow diet (A_CD, pink), ApoE^-/-^ mice with the high-fat diet (A_HD, red), wild-type mice with the chow diet (W_CD, light blue), and wild-type mice with the high-fat diet (W_HD, dark blue), displayed the particle numbers and Apo-B in plasma. (**A**) Particle number (PN) in plasma of the total Apo-B-carrying particles (TBPN) and in LDL (LDPN). (**B**) Apo-B in total plasma (TPAB) and in LDL (LDAB). (**C**) PN in the LDL subclass (L1PN-L6PN). (**D**) Apo-B in the LDL subclass (L1AB-L6AB). The asterisks indicate the levels of significance in the same types of mice with different diets. One asterisk (*) indicates the *p*-value < 0.05; three asterisks (***) indicate the *p*-value < 0.001; and ns indicate not significant.

NMR-based lipoprotein subclass analysis also provided information on particle numbers and Apo-B, in which two types of mice showed varied proportions. The bar charts display the total amount of Apo-B and different lipoprotein classes of the particle number (PN) and LDL subclass levels of four groups of mice. Wild-type mice did not express the significance of different lipoproteins in the PN and Apo-B. However, ApoE^-/-^ mice had higher significance in PN in the plasma of the total Apo-B-carrying particles (TBPN), in LDL (LDPN) (Figure 5A) and Apo-B in the total plasma (TPAB), and in LDL (LDAB) (Figure 5B).

Furthermore, the linear charts provide thorough information on diverse distributions of PN and the amount of Apo-B in different LDL subclasses. In the PN of the lipoprotein classes linear chart, wild-type mice had a substantial difference in L5PN (Figure 5B). On the other hand, even though ApoE^-/-^ mice had less significance in L1PN (Figure 5B), they had high significance in L2PN, L3PN, and L4PN. Apo-B presented the same trend of PN.

## 3. Discussion

Previous studies have documented that lipid accumulation increases the risk of cardiovascular diseases, where the new testing of lipoprotein profiles has already been applied to research phases due to advanced technologies, including NMR with its post-analysis software and in vitro diagnostic for research in the lipoprotein subclass analysis platform. Through a comprehensive examination of 112 lipoprotein fractions and component plasma levels in mice treated with high-fat diets, we found profound alterations in TG, CH, FC, and PL levels in the LDL fraction in ApoE^-/-^ mice and dramatic changes in CH, FC, and PL levels in HDL in wild-type mice as well. The proportions of TG in wild-type mice and CH, FC, and PL in ApoE^-/-^ mice showed apparent variations in lipoproteins (such as VLDL, LDL, IDL, and HDL) (Figure 2). Furthermore, the subfractions of each lipoprotein demonstrated the distributions of TG, CH, FC, and PL in four groups of mice, which showed different patterns (Figure 4). This is the first study that explores the distribution and composition of lipoprotein on ApoE^-/-^ mice with a high-fat diet using an NMR spectrometer (Bruker Biospin GmbH, Rheinstetten, Germany). The thorough compositional analyses expand the realm of lipoprotein subfractions and explain the level and distribution of the condition, which is affected by the APOE protein and high-fat diet in mice models.

**Figure 6:**
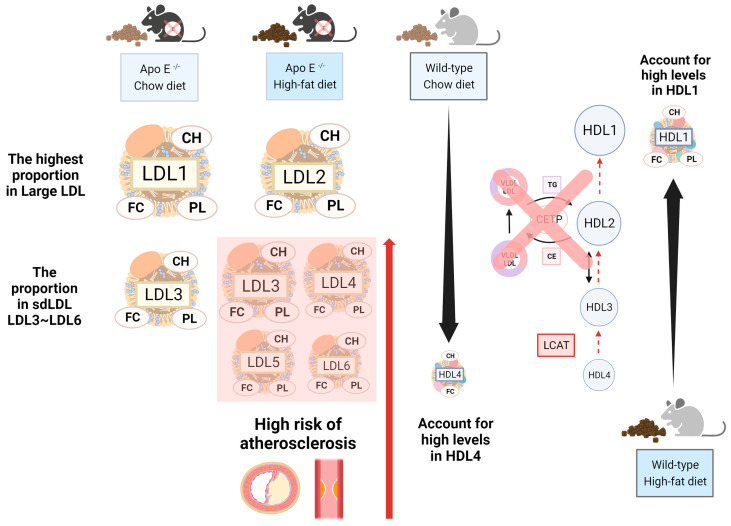
The highlighted alterations of the lipoprotein profile in ApoE-deficient mice and wild-type mice. There were four groups of mice: ApoE^-/-^ mice with the chow diet, ApoE^-/-^ mice with the high-fat diet, wild-type mice with the chow diet, and wild-type mice with high-fat diets. Even though ApoE^-/-^ mice had large amounts of LDL, the group with chow diets and high-fat diets had CH, FC, and PL in different LDL subfractions. We observed that the group with high-fat diets had a higher amount of sdLDL than the group with chow diets, which may lead to an elevated risk of atherosclerosis. On the other hand, wild-type mice had large amounts of HDL but had different subfractions in the group with chow diets and high-fat diets. Since we understand mice lacking CETP and the lower LCAT rate in the reverse cholesterol transport pathway, in this situation, we observed that the group with chow diets had the largest amounts of CH and FC in HDL4, while the group with high-fat diets had the highest amounts of CH, FC, and PL in HDL1.

The wild-type group with chow diets had large amounts of TG in VLDL, while the other three components, including CH, FC, and PL, took up large amounts in HDL, due to the deficiency of the cholesteryl ester transfer protein (CETP), a protein that shuffles CH between HDL and LDL in humans [22]. For this reason, most CHs are carried in HDL in wild-type mice [3]. We propose that the conversion from HDL4 to HDL1 decreases due to the lower lecithin cholesterol acyl transferase (LCAT) rate. Therefore, the number of subfractions decreases by the order in the HDL subclass, i.e., HDL4 and HDL1 will have the highest and the lowest percentages, respectively. We observed this phenomenon in CH and FC in wild-type mice (Figure 4). On the other hand, the distribution of the group of wild-type mice with the high-fat diet was the opposite; those mice showed that the number of subfractions increased by the order in the HDL subclass, where the concentration of HDL1 was the highest (Figure 3) and accounted for the most significant proportion in the HDL subclass, with it being more than 10% higher than the other HDL subfractions (Figure 4). We hypothesized that (Figure 6), in addition to excessive fat intake that may promote the entire reverse cholesterol transport (RCT) pathway, HDL-1 has a larger particle size and carries Apo-A1 with a large amount of Apo-E, which can promote LCAT activation. Furthermore, two groups of wild-type mice had slightly more FC in LDL than in HDL. Currently, we do not have an explanation because of the insufficient number of samples, and therefore, we will have to conduct more extensive research in the future.

Furthermore, we observed that in ApoE^-/-^ mice, except for TG, which had a high proportion of VLDL, the other three components, CH, FC, and PL, had a high proportion of LDL. The group with chow diets had the highest proportion of LDL-1, where the number of subfractions decreased by the order in the LDL subclass. However, the group with high-fat diets showed the highest proportion of LDL-2 in the LDL subclass. On the other hand, our study presented LDL3~LDL6 as sdLDL in the LDL subclass profile, and we observed that the group with high-fat diets had a more significant proportion of CH, FC, and PL in LDL3~LDL6 than the group with chow diets (Figure 4); the group with chow diets only took a small amount percentage in LDL3 (Supplementary Table S2). Most findings regarded LDL-1 and LDL-2 as protective in the absence of or with only trace concentrations of sdLDL, which is considered non-atherogenic to improve the ability against cardiovascular disease [23]. Therefore, we suggest that the mice that took high-fat diets had a higher risk of cardiovascular disease from the result.

The compositions and proportions of each lipid on the lipoproteins, such as TG, CH, FC, and PL, affect the density of lipoprotein subclasses. Although ultracentrifugation is considered the golden standard method in lipoprotein subclass analysis, it is time consuming and difficult to reach the requirement for clinical application. New testing for extended blood lipids and lipoproteins analysis are essential. Currently, the NMR-based method enables the routine blood testing of lipoproteins and adopts the algorithm of regression model for as close a value of ultracentrifugation as possible [24]. Herein, we use low-volume plasma to demonstrate the difference in lipids in lipoproteins of high-fat-treated mice. Meanwhile, the level and distribution of lipids in 15 lipoprotein subclasses were illustrated. It is comprehensive to distinguish the diet’s effect on blood lipids and their association with genetic background.

There are some limitations in this study. First, it is crucial to utilize fresh samples when identifying lipoprotein subclasses, as the freezing and thawing process can potentially lead to changes in the characteristics of lipoproteins. Specifically, it has been observed that freezing and thawing can result in a decrease in the size of HDL particles, a decrease in large LDL, and an increase in IDL [25,26]. Therefore, to obtain accurate and reliable information about lipoprotein subclasses, it is important to avoid the freezing and thawing of samples and to work with fresh specimens. Second, although the function of Apo-E is involved in removing atherogenic remnant lipoproteins from plasma and reducing foam cell formation in the vessel wall [27], it is an important animal model for the study of atherosclerosis [3]. However, the deficiency of CETP in ApoE^-/-^ mice will affect the LCAT turnover rate. It will possibly cause the RCT pathway to be different between humans and mice. The plasma lipoproteins profile might not be exactly the same as humans, even though we demonstrated profound alterations in TG, CH, FC, and PL levels of the LDL fraction in ApoE^-/-^ mice and CH, FC, and PL levels in HDL in wild-type mice when treated with a high-fat diet. Human CETP transgenic mice might be a well-established model for studying lipoprotein metabolism [3,28,29]. Moreover, another limitation is that the regression model of NMR in vitro diagnostic for research in the lipoprotein subclass analysis platform was established by human plasma [24], not mice. The accuracy of 112 lipoprotein fractions in our study may be reduced. By scaling up the mouse sample size, we may establish more reliable and robust results, enhancing the quality and precision of the lipoprotein fraction identification platform.

Interestingly, most CHs carried in HDL in wild-type mice were similar to what was previously reported in mice, due to their deficiency in CETP [22]. It would be fascinating to study the effects of the high-fat diet on plasma lipoproteins and whether the Apo-E protein has a protective effect on plasma CH reduction and prevents atherosclerosis formation. Future studies could establish a mice-specific regression model to improve lipoprotein subfraction assessment accuracy.

Despite previous clinical and cross-sectional studies that have found a positive relationship between higher levels of LDL particles and cardiovascular disease [30,31,32], the detailed comparison of compositional proportions and concentrations of different components within LDL particles of varying sizes has not been thoroughly explored. Examining the distribution and compositional characteristics of CH, TG, PL, and other lipid components within LDL particles of various sizes will contribute to a deeper understanding of their potential roles in the development and progression of cardiovascular disease. This investigation can shed light on mechanistic pathways associated with cardiovascular disease.

## 4. Materials and Methods

### 4.1. Animals

The Institutional Animal Care and Use Committee (IACUC) of Chang Gung University (CGU) approved these experiments (CGU107-269 and CGU108-163), and all tests were conducted in compliance with the institution’s regulations. Humane endpoints were used, and ethical principles were adhered to throughout the study.

The eight-week-old male C57BL/6JNarl (wild-type) and C57BL/6-Apoeem1Narl/Narl (ApoE^−/−^) mice were provided by the National Laboratory Animal Center (NLAC), NARLabs, Taiwan. The animals were kept in specific pathogen-free environments and were sacrificed when they were 20-weeks old. Following this, animals were divided into four groups. Group I—C57BL/6 wild-type (*n* = 5)—was fed with the chow diet (D12450K; Research Diets Inc., New Brunswick, NJ, USA), Group II—ApoE−/− (*n* = 4)—was fed with the chow diet, Group III—wild-type (*n* = 5)—was fed with the high-fat/high-sucrose diet (D12079B; Research Diets Inc., New Brunswick, NJ, USA), and Group IV—ApoE−/− (*n* = 4)—was fed with the high-fat/high-sucrose diet. The high-fat/high-sucrose diet was procured from Research Diets Inc. and comprised 17% kcal protein, 40% kcal fat, and 43% kcal carbohydrates per 100 g of pellet. Mice were sacrificed with cardiac puncture under isoflurane anesthesia when the mice were 21 weeks old. The plasma separated from whole blood was drawn into anti-coagulated (K2-EDTA) collection tubes by centrifugation for 10 min at 800× *g* at room temperature. After centrifugation, plasma was transferred and prepared for NMR measurement.

### 4.2. ^1^H Nuclear Magnetic Resonance Analysis

The 100 μL plasma supernatant was mixed with 75 mM, pH 7.4 sodium phosphate (buffer in 1:1 ratio), and 200 μL were transferred into a 3 mm × 4 inch Bruker SampleJet NMR tube (Bruker Biospin GmbH, Rheinstetten, Germany) [33]. All NMR analyses were completed on a Bruker Avance III HD 600 MHz spectrometer equipped with TXI probes and the Bruker SampleJet robot cooling system set to 6 °C (Bruker Biospin GmbH, Rheinstetten, Germany). For each blood sample, two experiments were completed in automation: first, an ^1^H 1D experiment with solvent presaturation (64 scans) and second, a 1D Carr–Purcell–Meiboom–Gill (CPMG) spin–echo experiment (64 scans). All data were processed in automation using Bruker Topspin 3.6.2 and ICON NMR (Bruker Biospin GmbH, Rheinstetten, Germany) for phasing, baseline correction, and calibration (TSP to 0 ppm). Lipoprotein reports containing 112 lipoprotein parameters (Supplementary Table S1) for each sample were generated using the Bruker IVDr lipoprotein subclass analysis (B.I.-LISA) method. This was completed by mathematically interrogating and quantifying the −CH_2_ (δ = 1.25 ppm) and −CH_3_ (δ = 0.8 ppm) peaks of the 1D spectrum after normalization to the Bruker QuantRef manager within Topspin using a PLS-2 regression model [24]. The lipoprotein data described chemical components of CH, FC, TGs, PLs, Apo-A1, Apo-A2, Apo-B, and Apo-B100 in different density classes: HDL (1.063–1.210 kg/L), IDL (1.006–1.019 kg/L), LDL (1.019–1.63 kg/L), and VLDL (0.950–1.006 kg/L). There were six LDL subfraction subclasses (LDL-1: 1.019–1.031 kg/L, LDL-2: 1.031–1.034 kg/L, LDL-3: 1.034–1.037 kg/L, LDL-4: 1.037–1.040 kg/L, LDL-5: 1.040–1.044 kg/L, and LDL-6: 1.044–1.063 kg/L), four HDL subfraction classes (HDL-1: 1.063–1.100 kg/L, HDL-2: 1.100–1.112 kg/L, HDL-3: 1.112–1.125 kg/L, and HDL-4: 1.125–1.210 kg/L), and five VLDL subfraction subclasses [34].

### 4.3. Statistics

To analyze NMR-based metabolomic data using the MetaboAnalyst software 6.0 package [35,36], we performed principal component analysis (PCA) using the concentrations of lipoprotein subfractions that were normalized and log-transformed. We used partial least squares discriminant analysis (PLS-DA) to analyze the results. We also performed Spearman’s correlation coefficients analysis to assess the associations between lipoprotein subfractions and diet. All data were expressed as mean ± standard deviation (mean ± SD) or percentage (%) as appropriate. Data were analyzed using GraphPad Prism 8. Most data were analyzed using two-way ANOVAs (APOE × diet), with Bonferroni post hoc tests where applicable. The student t-test was applied for the comparison of the chow diet and the high-fat diet within the same mice strain. Comparisons with *p* < 0.05 were considered statistically significant.

## 5. Conclusions

By utilizing NMR technology, along with its application software in vitro diagnostic for research in the lipoprotein subclass analysis platform, we are able to acquire lipoprotein profiles and generate quantitative data on various lipoprotein subfractions and their constituents in animal studies. This methodology facilitates a comprehensive study of the distribution and composition of different lipoprotein subfractions, providing valuable insights into their associations with cardiovascular outcomes.

## Data Availability

The data presented in this study are available upon request.

## Supplementary Materials

The following supporting information can be downloaded at: https://www.mdpi.com/article/10.3390/molecules29050988/s1, Figure S1: Body weight of chow diet or high-fat/high-sucrose diet-fed mice; Table S1: List of the 112 lipoprotein measured parameters, including 10 parameters which are calculated from the original ones. Table S2: The comparison of cholesterol, free cholesterol, and phospholipid in the sum of LDL3~LDL6, respectively, between the group of high-fat diets (A_CD) and the group of high-fat diets (A_HD).
