# Supplementary material for: NMR-Based Analysis of Plasma Lipoprotein Subclass and Lipid Composition Demonstrate the Different Dietary Effects in ApoE-Deficient Mice
Source: Molecules. 2024 Feb 24;29(5):988. doi: 10.3390/molecules29050988 (PMC10934052; doi:10.3390/molecules29050988)
Supplement: Supplementary file 1 [file molecules-29-00988-s001.zip › molecules-2889528-supplementary.pdf]

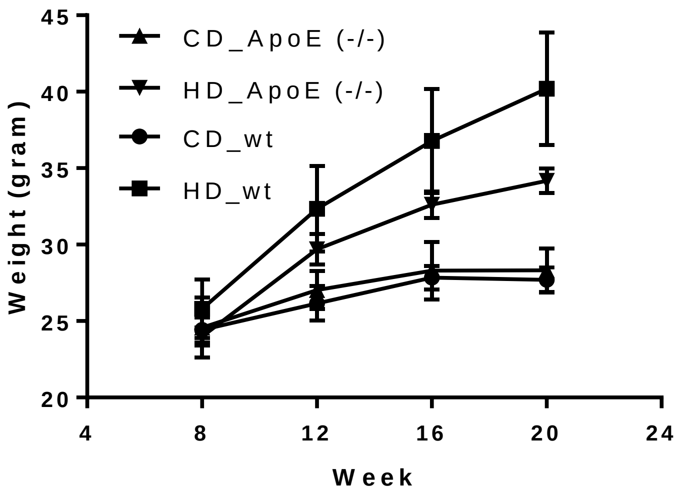

Supplementary Figure S1. Body weight of chow diet or high-fat/high-sucrose diet fed mice.
Mice body weight (g) were measured every four weeks. Data are expressed as the mean ±SD. CD, chow diet. HD, high-fat/high-sucrose diet.

Supplementary Table S1. List of the 112 lipoprotein measured parameters including 10 parameters which are calculated from the original ones.

Parameter	Comment	A_CD	A_HD	W_CD	W_HD
TPTG	Main Parameters, Triglycerides, TG	339.32±72	284.47±58	67.02±13	64.57±13
TPCH	Main Parameters, Cholesterol, Chol	363.76±49	626.11±106	117.17±5	187.28±28
LDCH	Main Parameters, LDL Cholesterol, LDL-Chol	80.19±14	376.93±40	28.04±6	52.67±20
HDCH	Main Parameters, HDL Cholesterol, HDL-Chol	19.82±3	46.39±6	89.61±2	130.14±12
TPA1	Main Parameters, Apo-A1, Apo-A1	107.74±8	305.83±37	144.82±5	187.38±15
TPA2	Main Parameters, Apo-A2, Apo-A2	18.56±2	54.19±7	51.36±5	29.04±6
TPAB	Main Parameters, Apo-B100, Apo-B100	120.49±11	267.17±31	35.27±4	42.58±11
TBPN	Calculated Figures, Total ApoB Particle Number, Total Particle Number	2190.80±196	4857.83±569	641.23±67	774.28±207
VLPN	Calculated Figures, VLDL Particle Number, VLDL Particle Number	523.31±106	314.14±74	25.67±8	1.28±3
IDPN	Calculated Figures, IDL Particle Number, IDL Particle Number	240.02±49	290.31±65	0.00±0	0.00±0
LDPN	Calculated Figures, LDL Particle Number, LDL Particle Number	799.88±70	2981.73±260	662.54±60	850.66±221
L1PN	Calculated Figures, LDL-1 Particle Number, LDL-1 Particle Number	666.16±109	1000.52±234	4.69±7	157.61±58
L2PN	Calculated Figures, LDL-2 Particle Number, LDL-2 Particle Number	626.06±75	1665.90±221	1.79±3	229.70±88
L3PN	Calculated Figures, LDL-3 Particle Number, LDL-3 Particle Number	214.00±94	1321.72±165	0.00±0	0.00±0
L4PN	Calculated Figures, LDL-4 Particle Number, LDL-4 Particle Number	0.00±0	313.55±80	0.00±0	0.00±0
L5PN	Calculated Figures, LDL-5 Particle Number, LDL-5 Particle Number	0.00±0	0.00±0	118.44±61	0.00±0
L6PN	Calculated Figures, LDL-6 Particle Number, LDL-6 Particle Number	57.03±114	0.00±0	588.53±29	580.25±47
VLTG	Lipoprotein Main Fractions, Triglycerides, VLDL	123.55±34	111.38±18	60.81±10	11.46±11
IDTG	Lipoprotein Main Fractions, Triglycerides, IDL	39.20±11	27.35±9	5.99±2	6.39±3
LDTG	Lipoprotein Main Fractions, Triglycerides, LDL	43.09±6	71.27±10	0.00±0	8.17±3
HDTG	Lipoprotein Main Fractions, Triglycerides, HDL	18.79±2	28.71±3	0.74±1	2.66±2
VLCH	Lipoprotein Main Fractions, Cholesterol, VLDL	155.21±32	109.81±31	0.00±0	1.21±1
IDCH	Lipoprotein Main Fractions, Cholesterol, IDL	69.77±12	97.60±21	0.00±0	0.00±0

LDCH	Lipoprotein Main Fractions, Cholesterol, LDL	80.19±14	376.93±40	28.04±6	52.67±20
HDCH	Lipoprotein Main Fractions, Cholesterol, HDL	19.82±3	46.39±6	89.61±2	130.14±12
VLFC	Lipoprotein Main Fractions, Free Cholesterol, VLDL	42.65±9	26.61±7	4.22±1	3.20±0
IDFC	Lipoprotein Main Fractions, Free Cholesterol, IDL	18.30±3	25.24±6	0.00±0	0.00±0
LDFC	Lipoprotein Main Fractions, Free Cholesterol, LDL	26.87±4	128.64±15	10.41±1	16.66±5
HDFC	Lipoprotein Main Fractions, Free Cholesterol, HDL	18.26±1	46.55±6	19.54±1	32.18±3
VLPL	Lipoprotein Main Fractions, Phospholipids, VLDL	35.52±11	0.00±0	8.81±1	1.67±1
IDPL	Lipoprotein Main Fractions, Phospholipids, IDL	18.66±5	19.77±5	0.00±0	0.40±1
LDPL	Lipoprotein Main Fractions, Phospholipids, LDL	42.52±5	160.10±18	21.45±2	34.78±9
HDPL	Lipoprotein Main Fractions, Phospholipids, HDL	27.49±6	16.99±5	120.60±4	161.62±14
HDA1	Lipoprotein Main Fractions, Apo-A1, HDL	117.42±8	291.68±38	158.60±5	210.88±18
HDA2	Lipoprotein Main Fractions, Apo-A2, HDL	17.40±2	48.27±5	53.67±4	31.28±6
VLAB	Lipoprotein Main Fractions, Apo-B, VLDL	28.78±6	17.28±4	1.41±0	0.07±0
IDAB	Lipoprotein Main Fractions, Apo-B, IDL	13.20±3	15.97±4	0.00±0	0.00±0
LDAB	Lipoprotein Main Fractions, Apo-B, LDL	43.99±4	163.99±14	36.44±3	46.79±12
V1TG	VLDL Subfractions, Triglycerides, VLDL-1	0.00±0	0.00±0	58.45±11	3.91±7
V2TG	VLDL Subfractions, Triglycerides, VLDL-2	55.23±14	51.95±12	0.05±0	1.24±1
V3TG	VLDL Subfractions, Triglycerides, VLDL-3	62.28±13	66.29±14	0.00±0	0.14±0
V4TG	VLDL Subfractions, Triglycerides, VLDL-4	26.40±5	26.37±5	2.59±1	2.21±1
V5TG	VLDL Subfractions, Triglycerides, VLDL-5	5.10±1	0.02±0	6.18±1	6.46±0
V1CH	VLDL Subfractions, Cholesterol, VLDL-1	40.68±9	20.16±6	4.29±1	3.75±1
V2CH	VLDL Subfractions, Cholesterol, VLDL-2	28.45±6	20.68±6	0.00±0	1.08±1
V3CH	VLDL Subfractions, Cholesterol, VLDL-3	30.43±6	26.50±7	0.00±0	0.00±0
V4CH	VLDL Subfractions, Cholesterol, VLDL-4	33.39±7	20.77±7	0.00±0	0.00±0
V5CH	VLDL Subfractions, Cholesterol, VLDL-5	3.45±1	0.00±0	2.96±0	3.24±0

V1FC	VLDL Subfractions, Free Cholesterol, VLDL-1	2.70±1	0.00±0	0.10±0	0.00±0
V2FC	VLDL Subfractions, Free Cholesterol, VLDL-2	13.19±3	12.90±3	0.00±0	0.00±0
V3FC	VLDL Subfractions, Free Cholesterol, VLDL-3	10.50±2	9.14±2	0.00±0	0.00±0
V4FC	VLDL Subfractions, Free Cholesterol, VLDL-4	14.99±3	15.76±4	0.00±0	0.00±0
V5FC	VLDL Subfractions, Free Cholesterol, VLDL-5	1.62±1	0.00±0	1.86±0	1.95±0
V1PL	VLDL Subfractions, Phospholipids, VLDL-1	4.63±3	0.00±0	4.18±1	0.00±0
V2PL	VLDL Subfractions, Phospholipids, VLDL-2	13.86±4	8.53±2	0.00±0	0.00±0
V3PL	VLDL Subfractions, Phospholipids, VLDL-3	14.84±4	11.94±3	0.00±0	0.00±0
V4PL	VLDL Subfractions, Phospholipids, VLDL-4	19.59±4	14.21±4	0.00±0	0.00±0
V5PL	VLDL Subfractions, Phospholipids, VLDL-5	4.04±1	0.00±0	3.16±1	3.26±0
L1TG	LDL Subfractions, Triglycerides, LDL-1	23.92±3	37.32±8	0.00±0	0.26±0
L2TG	LDL Subfractions, Triglycerides, LDL-2	3.71±1	10.73±2	0.00±0	0.56±1
L3TG	LDL Subfractions, Triglycerides, LDL-3	1.29±0	0.04±0	1.84±0	2.66±0
L4TG	LDL Subfractions, Triglycerides, LDL-4	1.92±1	1.52±1	0.00±0	0.00±0
L5TG	LDL Subfractions, Triglycerides, LDL-5	0.20±0	0.00±0	0.17±0	0.96±1
L6TG	LDL Subfractions, Triglycerides, LDL-6	3.57±2	0.00±0	5.72±0	6.53±1
L1CH	LDL Subfractions, Cholesterol, LDL-1	74.21±13	108.52±27	0.00±0	15.44±7
L2CH	LDL Subfractions, Cholesterol, LDL-2	61.32±8	165.48±22	0.34±0	27.69±10
L3CH	LDL Subfractions, Cholesterol, LDL-3	23.42±9	138.62±18	0.00±0	0.00±0
L4CH	LDL Subfractions, Cholesterol, LDL-4	0.00±0	28.42±8	0.00±0	0.00±0
L5CH	LDL Subfractions, Cholesterol, LDL-5	0.00±0	0.00±0	9.06±5	0.00±0
L6CH	LDL Subfractions, Cholesterol, LDL-6	0.00±0	0.00±0	38.60±2	37.48±3
L1FC	LDL Subfractions, Free Cholesterol, LDL-1	24.73±4	43.04±9	0.00±0	4.49±2
L2FC	LDL Subfractions, Free Cholesterol, LDL-2	22.96±3	61.04±8	1.43±1	10.01±3
L3FC	LDL Subfractions, Free Cholesterol, LDL-3	8.36±2	43.93±6	2.77±0	6.28±1

L4FC	LDL Subfractions, Free Cholesterol, LDL-4	0.00±0	18.99±2	1.10±1	0.00±0
L5FC	LDL Subfractions, Free Cholesterol, LDL-5	0.00±0	0.00±0	4.23±1	1.86±1
L6FC	LDL Subfractions, Free Cholesterol, LDL-6	0.13±0	4.13±2	9.79±0	9.06±1
L1PL	LDL Subfractions, Phospholipids, LDL-1	39.82±7	53.64±14	0.00±0	8.73±3
L2PL	LDL Subfractions, Phospholipids, LDL-2	33.17±4	84.29±12	0.53±1	14.89±4
L3PL	LDL Subfractions, Phospholipids, LDL-3	13.41±4	67.78±9	0.00±0	0.38±0
L4PL	LDL Subfractions, Phospholipids, LDL-4	0.00±0	12.74±4	0.00±0	0.00±0
L5PL	LDL Subfractions, Phospholipids, LDL-5	0.00±0	0.00±0	4.90±3	0.07±0
L6PL	LDL Subfractions, Phospholipids, LDL-6	0.00±0	0.00±0	22.18±1	23.01±2
L1AB	LDL Subfractions, Apo-B, LDL-1	36.64±6	55.03±13	0.26±0	8.67±3
L2AB	LDL Subfractions, Apo-B, LDL-2	34.43±4	91.62±12	0.10±0	12.63±5
L3AB	LDL Subfractions, Apo-B, LDL-3	11.77±5	72.69±9	0.00±0	0.00±0
L4AB	LDL Subfractions, Apo-B, LDL-4	0.00±0	17.24±4	0.00±0	0.00±0
L5AB	LDL Subfractions, Apo-B, LDL-5	0.00±0	0.00±0	6.51±3	0.00±0
L6AB	LDL Subfractions, Apo-B, LDL-6	3.14±6	0.00±0	32.37±2	31.91±3
H1TG	HDL Subfractions, Triglycerides, HDL-1	2.65±0	10.46±1	0.00±0	3.66±2
H2TG	HDL Subfractions, Triglycerides, HDL-2	4.50±0	7.98±1	0.79±1	2.17±1
H3TG	HDL Subfractions, Triglycerides, HDL-3	4.95±0	7.57±1	1.16±1	1.25±0
H4TG	HDL Subfractions, Triglycerides, HDL-4	6.11±1	2.63±1	0.00±0	0.00±0
H1CH	HDL Subfractions, Cholesterol, HDL-1	0.00±0	3.54±3	16.73±4	60.10±11
H2CH	HDL Subfractions, Cholesterol, HDL-2	3.27±1	4.10±1	20.67±2	32.71±3
H3CH	HDL Subfractions, Cholesterol, HDL-3	5.20±1	14.11±1	22.67±1	22.80±2
H4CH	HDL Subfractions, Cholesterol, HDL-4	16.62±4	30.94±8	24.93±7	5.67±4
H1FC	HDL Subfractions, Free Cholesterol, HDL-1	0.21±0	12.95±1	4.83±1	12.81±2
H2FC	HDL Subfractions, Free Cholesterol, HDL-2	2.33±0	7.97±1	4.69±0	5.34±0

H3FC	HDL Subfractions, Free Cholesterol, HDL-3	3.31±0	12.37±2	4.76±0	3.08±0
H4FC	HDL Subfractions, Free Cholesterol, HDL-4	6.68±1	18.24±3	6.58±2	0.62±1
H1PL	HDL Subfractions, Phospholipids, HDL-1	0.00±0	0.00±0	22.05±5	70.61±12
H2PL	HDL Subfractions, Phospholipids, HDL-2	3.33±1	0.00±0	31.79±3	45.19±4
H3PL	HDL Subfractions, Phospholipids, HDL-3	4.61±1	2.79±0	35.32±3	33.54±3
H4PL	HDL Subfractions, Phospholipids, HDL-4	17.84±5	11.11±7	34.66±9	9.68±6
H1A1	HDL Subfractions, Apo-A1, HDL-1	0.00±0	26.49±2	17.12±12	100.06±22
H2A1	HDL Subfractions, Apo-A1, HDL-2	0.00±0	0.00±0	29.18±1	35.01±3
H3A1	HDL Subfractions, Apo-A1, HDL-3	21.02±1	44.66±5	51.38±3	48.32±4
H4A1	HDL Subfractions, Apo-A1, HDL-4	83.67±11	167.62±33	60.86±19	10.41±8
H1A2	HDL Subfractions, Apo-A2, HDL-1	0.00±0	0.00±0	4.03±1	7.92±1
H2A2	HDL Subfractions, Apo-A2, HDL-2	0.00±0	0.00±0	6.85±0	7.18±1
H3A2	HDL Subfractions, Apo-A2, HDL-3	1.92±1	5.81±0	13.44±1	9.91±1
H4A2	HDL Subfractions, Apo-A2, HDL-4	15.90±3	33.31±7	24.97±6	1.16±2

The concentration unit of main parameters, lipoprotein fractions, and lipoprotein subfractions are mg/dL, and the unit of calculated figures of Particle Number, including TBPN, IDPN, LDPN, L1PN, L2PN, L3PN, L4PN, L5PN, and L6PN, are nmol/L.

Supplementary Table S2. The comparison of cholesterol, free cholesterol, and phospholipid in the sum of LDL3~LDL6, respectively, between the group of high-fat diets (A_CD) and the group of high-fat diets (A_HD)

Component / The sum of LDL3~LDL6	The group of high-fat diets (A_CD)	The group of high-fat diets (A_HD)
Cholesterol (Chol)	7.31% (only LDL3)	28.70%
Free cholesterol (FC)	7.48% (only LDL3)	24.16%
Phospholipid (PL)	7.93 % (only LDL3)	30.15%
